# Supplementary material for: Integrative multi-omic sequencing reveals the MMTV-Myc mouse model mimics human breast cancer heterogeneity
Source: Breast Cancer Res. 2023 Oct 7;25:120. doi: 10.1186/s13058-023-01723-3 (PMC10559619; doi:10.1186/s13058-023-01723-3)

Consensus NNNNNNNNNNYCCNCTTCTTTACTTTGCATTTAAAGAKCACATCCARGMCCACAYTCTG- 59

222-1_EMT_6_KIT-KIT_F_R.ab1 --........CNN.NNN.NN.NNN.............G........G.A.....C....- 57

525-1_Squamous_9_KIT-KIT_F_R.ab1 ---.......N.N...N.N..................G........G.A.....C....- 56

598-1_Squamous_10_KIT-KIT_F_R.ab1 ------....NN..N.N...N................G........G.A.....C....- 53

642-1_EMT_14_KIT-KIT_F_R.ab1 ---....A..N.N.N.....N................G........G.A.....C....- 56

812-1_EMT_7_KIT-KIT_F_R.ab1 ---G......N.NC......N................G........G.A.....C....- 56

854-2_Microac_1_KIT-KIT_F_R.ab1 -----.....NT..N.....N................G........G.C.....C....- 54

864-1_Microac_2_KIT-KIT_F_R.ab1 ----G.....T.NC......NN...............G........G.A.....C....- 55

1052-1_Microac_3_KIT-KIT_F_R.ab1 ----......NT..N......................G........G.A.....C....G 56

1062-1_Microac_4_KIT-KIT_F_R.ab1 ----....A.NN..N......................G........G.A.....C....G 56

1066-1_Squamous_11_KIT-KIT_F_R.ab1 ---...A...NNNC..................-N...G........G.A.....C....- 55

1066-2_Squamous_12_KIT-KIT_F_R.ab1 ------....NNN.N......................G..-.....G.A.....C....- 52

1139-1_EMT_15_KIT-KIT_F_R.ab1 ---.......N.G.N......................G........G.C.....C....- 56

1356-2_EMT_8_KIT-KIT_F_R.ab1 .........TNNNC......N...........-....G........G.C.....C....- 58

1445-1_Squamous_13_KIT-KIT_F_R.ab1 -----G....N.GC......N................G..-.....G.C.....C....- 53

1576-1_Microac_5_KIT-KIT_F_R.ab1 ----......T.GCN......................G........G.A.....C....- 55

Consensus YTCACRCYKCTGCTCMTYGGCTKKSYSKTSNCAKYTGGCGCGATGGKGATCATTRTGATG 119

222-1_EMT_6_KIT-KIT_F_R.ab1 T....G.CG......A.T....TTGTGG.C...GC...........G.......G..... 117

525-1_Squamous_9_KIT-KIT_F_R.ab1 T....G.CG......A.T....TTGNGG.C...GC.....A.....G.......G..... 116

598-1_Squamous_10_KIT-KIT_F_R.ab1 T....G.CG......A.T....TTGTGG.C...GC.....A.....G.......G..... 113

642-1_EMT_14_KIT-KIT_F_R.ab1 T....G.CG......A.T....TTGTGG.C...GC...........G.......G..... 116

812-1_EMT_7_KIT-KIT_F_R.ab1 T....G.CG......A.T....TTGTGG.C...GC...........G.......G..... 116

854-2_Microac_1_KIT-KIT_F_R.ab1 T....G.CG......A.T....TTGTGG.C...GC...........G.......G..... 114

864-1_Microac_2_KIT-KIT_F_R.ab1 T..N.G.CG......A.T....TTGTGG.C...GC.....A.....G.......G..... 115

1052-1_Microac_3_KIT-KIT_F_R.ab1 T..C.G.CG......A.T....TTGTGG.C...GC...NNN.....G.......G..... 116

1062-1_Microac_4_KIT-KIT_F_R.ab1 T..N.G.CG......A.T....TTGTGG.C...TC...........G.......G..... 116

1066-1_Squamous_11_KIT-KIT_F_R.ab1 -NNN.G.CG......A.T....TTGTGG.C..NGC...........G.......G..... 114

1066-2_Squamous_12_KIT-KIT_F_R.ab1 -.TN.G.CG......A.T....TTGTGG.C...GC...........G.......G..... 111

1139-1_EMT_15_KIT-KIT_F_R.ab1 -.TN.G.CG......A.T....TTGTGG.C...GC...........G.......G..... 115

1356-2_EMT_8_KIT-KIT_F_R.ab1 T....G.CG......A.T....TTGTGG.CG..TC.....A.....G.......G..... 118

1445-1_Squamous_13_KIT-KIT_F_R.ab1 T....G.CG......A.T....TTGTGG.CG..GC.....A.....G.......G..... 113

1576-1_Microac_5_KIT-KIT_F_R.ab1 T....G.CG......A.T....TTGTGG.C...GC.....A.....G.......G..... 115

Consensus RYGYTCACCWASRARYWYTYSYASAAACMYWTGYATRAASYMCWRTGKAARGTYSWCGAG 179

222-1_EMT_6_KIT-KIT_F_R.ab1 GT.C.....T.CA.GTAT.TGC.C....CCA..T..G..GTA.AA..G..G..TGT.... 177

525-1_Squamous_9_KIT-KIT_F_R.ab1 GT.C.....T.CA.GTAT.TGC.C....CCA..C..G..GTA.AA..G..G..TGT.... 176

598-1_Squamous_10_KIT-KIT_F_R.ab1 GT.C.....T.CA.ATAT.TGC.C....CCA..T..G..GTA.AA..G..G..TGT.... 173

642-1_EMT_14_KIT-KIT_F_R.ab1 GT.C.....T.CA.ATAT.TGC.C....CCA..T..G..GTA.AA..G..G..TGT.... 176

812-1_EMT_7_KIT-KIT_F_R.ab1 GT.C.....T.CN.ATAT.TGC.C....CCA..T..G..GTA.AA..G..G..TGT.... 176

854-2_Microac_1_KIT-KIT_F_R.ab1 GT.C.....T.CA.ATAT.TGC.C....CCA..T..G..GTA.AA..G..G..TGT.... 174

864-1_Microac_2_KIT-KIT_F_R.ab1 GT.C.....T.CA.ATAT.TGC.C....CCA..T..G..GTA.AA..G..G..TGT.... 175

1052-1_Microac_3_KIT-KIT_F_R.ab1 GT.C.....T.CA.GTAT.TGC.C....CCA..T..G..GTA.AA..G..G..TGT.N.. 176

1062-1_Microac_4_KIT-KIT_F_R.ab1 GT.C.....T.CA.GTAT.TGC.C....CCA..T..G..GTA.AA..G..G..TGT.... 176

1066-1_Squamous_11_KIT-KIT_F_R.ab1 GT.C.....T.CA.GTAT.TGC.C....CCA..T..G..GTA.AA..G..G..TGT.N.. 174

1066-2_Squamous_12_KIT-KIT_F_R.ab1 GT.C.....T.CA.ATAT.TGC.C....CCA..T..G..GTA.AA..G..G..TGT.... 171

1139-1_EMT_15_KIT-KIT_F_R.ab1 GT.C.....T.CA.ATAT.TGC.C....CCA..T..G..GTA.AA..G..G..TGT.... 175

1356-2_EMT_8_KIT-KIT_F_R.ab1 GT.C.....T.CA.ATAT.TGC.C....CCA..T..G..NTA.TA..G..G..TGT.... 178

1445-1_Squamous_13_KIT-KIT_F_R.ab1 GT.C.....T.CA.ATAT.TGC.C....CCA..T..G..GTA.AA..G..G..TGT.... 173

1576-1_Microac_5_KIT-KIT_F_R.ab1 GT.C.....T.CA.GTAT.TGC.C....CCA..T..G..GTA.AA..G..G..TGT.... 175

Consensus RANRTARAYGRARACAWTTATGTWWACATWKAYSCKACGCWACWYCCTWMTSATCWCMMW 239

222-1_EMT_6_KIT-KIT_F_R.ab1 G.-NN.A.T.G.A...A......TT....AT.CC.G....A..TT...TA.G...A.CAA 236

525-1_Squamous_9_KIT-KIT_F_R.ab1 G..A..A.T.G.A...A......TT....AT.CC.G....A..TT...TA.G...A.CAA 236

598-1_Squamous_10_KIT-KIT_F_R.ab1 G..A..A.T.G.A...A......TT....AT.CC.G....A..TT...TA.G...T.CAA 233

642-1_EMT_14_KIT-KIT_F_R.ab1 G.-NN.A.T.G.A...A......TT....AT.CC.G....AN.TT...TA.G...A.CAA 235

812-1_EMT_7_KIT-KIT_F_R.ab1 G..A..A.T.G.A...A......TT....AT.CC.G....A..TT...TA.G...T.CAA 236

854-2_Microac_1_KIT-KIT_F_R.ab1 G..A..A.T.G.A...A......TT....AT.CC.G....A..TT...TA.G...T.CAA 234

864-1_Microac_2_KIT-KIT_F_R.ab1 G..A..A.T.G.A...A......TT....AT.CC.G....A..TT...TA.G...T.CAA 235

1052-1_Microac_3_KIT-KIT_F_R.ab1 G..A..A.T.G.A...A......TT....AT.CC.G....A..TT...TA.G...A.CCA 236

1062-1_Microac_4_KIT-KIT_F_R.ab1 G..A..A.T.G.A...A......TT....AT.CC.G....AN.TT...TA.G...A.CAA 236

1066-1_Squamous_11_KIT-KIT_F_R.ab1 G..A..A.T.G.A...A......TT....AT.CC.G....A..TT...TA.G...T.CAT 234

1066-2_Squamous_12_KIT-KIT_F_R.ab1 G..A..A.T.G.A...A......TT....AT.CC.G....A..TT...TA.G...A.AAA 231

1139-1_EMT_15_KIT-KIT_F_R.ab1 G..A..A.T.G.A...A......TT....AT.CC.G....A..TT...TA.G...A.AAA 235

1356-2_EMT_8_KIT-KIT_F_R.ab1 G..A..A.T.G.A...A......TT....AT.CC.G....A..TT...TA.G...A.ACA 238

1445-1_Squamous_13_KIT-KIT_F_R.ab1 G..A..A.T.G.A...A......TT....AT.CC.G....A..TT...TA.G...A.AAA 233

1576-1_Microac_5_KIT-KIT_F_R.ab1 G..A..A.T.G.A...A......TT....AT.CC.G....A..TT...TA.G...T.CAA 235

Consensus TGKGAKYTYCCCWGAAACWKGCTSAGWTTYGGAAWGACWTTGGGARCAACATTGGGAGCA 299

222-1_EMT_6_KIT-KIT_F_R.ab1 ..G..GT.T...A.....TG...G..T..T....A...A......G....N......... 296

525-1_Squamous_9_KIT-KIT_F_R.ab1 ..G..GT.T...A.....AG...G.NT..T....A...T......G....N......... 296

598-1_Squamous_10_KIT-KIT_F_R.ab1 ..G..GT.T...A.....AG...G..T..T....A...T......G....N......... 293

642-1_EMT_14_KIT-KIT_F_R.ab1 ..G..GT.T...A.....AG...G.NT..T....A...T......G.............. 295

812-1_EMT_7_KIT-KIT_F_R.ab1 ..G..GT.T...A.....AG...G..T..T....A...T......G.............. 296

854-2_Microac_1_KIT-KIT_F_R.ab1 ..G..GT.T...A.....AG...G..T..T....A...T......G.............. 294

864-1_Microac_2_KIT-KIT_F_R.ab1 ..G..GT.T...A.....AG...G..T..T....A...T......G.............. 295

1052-1_Microac_3_KIT-KIT_F_R.ab1 ..G..GT.T...A.....AT...G..T..T....A...T......G.............. 296

1062-1_Microac_4_KIT-KIT_F_R.ab1 ..G..GT.T...A.....AT...G.NT..T....A...T......G....N......... 296

1066-1_Squamous_11_KIT-KIT_F_R.ab1 ..G..GT.T...A.....TT...G..T..T....A...T......G.............. 294

1066-2_Squamous_12_KIT-KIT_F_R.ab1 ..G..GT.T...A.....AG...G..A..T....A...T......G.............. 291

1139-1_EMT_15_KIT-KIT_F_R.ab1 ..G..GT.T...A.....AG...G.NT..T....A...T......G....N......... 295

1356-2_EMT_8_KIT-KIT_F_R.ab1 ..G..GT.T...A.....AG...G.NT..T....A...A......G..........NNN- 297

1445-1_Squamous_13_KIT-KIT_F_R.ab1 ..G..GT.T...A.....AG...G..T..T....A...N......G..N-NN........ 292

1576-1_Microac_5_KIT-KIT_F_R.ab1 ..G..GT.T...A.....AG...G..T..T....A...T......G...-.......... 294

 
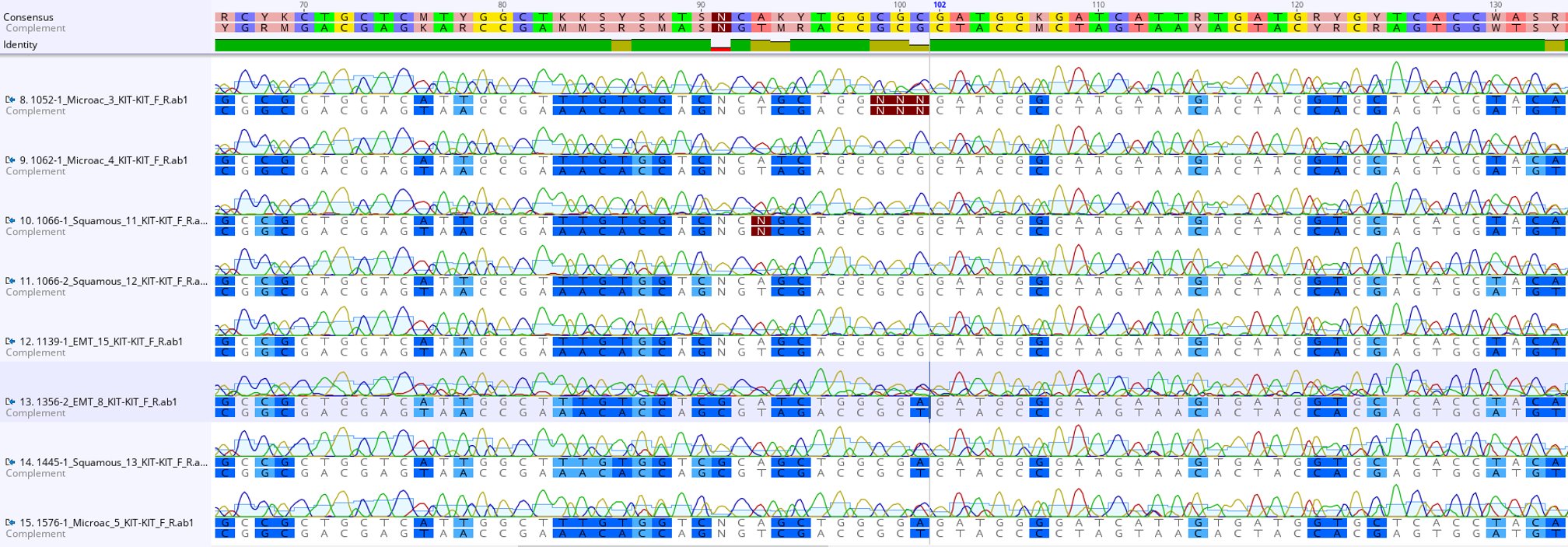

Supplement: Supplementary file 1 — Additional file 1: Aligned sanger sequencing results of 5 microacinar, 5 squamous, and 5 EMT tumors over KIT. Sequencing shows a conserved C to A mutation at consensus sequence position 100 and present in tumors 525-1, 598-1, 864-1, 1052-1, 1356-2, 1445-1, and 1576-1. An electropherogram of some sequences is included. [file 13058_2023_1723_MOESM1_ESM.docx]
