# Supplementary material for: Integrative multi-omic sequencing reveals the MMTV-Myc mouse model mimics human breast cancer heterogeneity
Source: Breast Cancer Res. 2023 Oct 7;25:120. doi: 10.1186/s13058-023-01723-3 (PMC10559619; doi:10.1186/s13058-023-01723-3)

Consensus NNNNNNNCRAGTCTYCCTGGA-MTTG--ACCTCTGGGACAA-GT--TCAGTGAACTCTCC 54

222-1_EMT_21_RARA-RARA_F_R.ab1 ----...NNNNNNNN.T.NNNNN.NNNN.TT.......A.--TC--C.....C....... 52

525-1_Squamous_24_RARA-RARA_F_R.ab1 .......NN....NNN-N...-A...--.............-..--.............. 53

598-1_Squamous_25_RARA-RARA_F_R.ab1 -.......N.....C......-A...--.............-..--.............. 53

642-1_EMT_29_RARA-RARA_F_R.ab1 -.......N.....C......CA...--.............-..--.............. 54

812-1_EMT_22_RARA-RARA_F_R.ab1 .......NN...NNC.-....-A...--.............-..--.............. 53

854-2_Microac_16_RARA-RARA_F_R.ab1 .......NN...NNN.-....CA...--.............-..--.............. 54

864-1_Microac_17_RARA-RARA_F_R.ab1 -......NN...N.N.-....-A...--.............-..--.............. 52

1052-1_Microac_18_RARA-RARA_F_R.ab1 --.....NNNNNNCTN...NN--.NNNC.TT.......A.--TC--......C....... 52

1062-1_Microac_19_RARA-RARA_F_R.ab1 -......NG....NC......-A...--.............-..--.............. 53

1066-1_Squamous_26_RARA-RARA_F_R.ab1 -......NN.....C......CA...--.............-..--.............. 54

1066-2_Squamous_27_RARA-RARA_F_R.ab1 --.....NN...NNNN-....C-...--.............-..--.............. 51

1139-1_EMT_30_RARA-RARA_F_R.ab1 -------NNNNNNNNNNNNNNNNNNNNNNNT.NNNNN.N.NANAAANNNNNNN.N.N.NN 53

1356-2_EMT_23_RARA-RARA_F_R.ab1 -......NN...NNN.-....-A...--.............-..--.............. 52

1445-1_Squamous_28_RARA-RARA_F_R.ab1 --......G.....C......-A...--.............-..--.............. 52

1576-1_Microac_20_RARA-RARA_F_R.ab1 ----...NANNNNNNN..NCNGNNNNNN.T........A.--..--......C....... 52

Consensus ACCAAGTGCATC-ATTAAGACTG-TGGAGTTCGCC-AA-GCAGCTTCCCGGCTTCACCAC 110

222-1_EMT_21_RARA-RARA_F_R.ab1 G....C......-...N.T....N.TT.....C..---AN.T............AT.... 108

525-1_Squamous_24_RARA-RARA_F_R.ab1 ............-..........-...........-..-..................... 109

598-1_Squamous_25_RARA-RARA_F_R.ab1 ............-..........-...........-..-..................... 109

642-1_EMT_29_RARA-RARA_F_R.ab1 ............-..........-...........-..-..................... 110

812-1_EMT_22_RARA-RARA_F_R.ab1 ............-..........-...........-..-..................... 109

854-2_Microac_16_RARA-RARA_F_R.ab1 ............-..........-...........-..-..................... 110

864-1_Microac_17_RARA-RARA_F_R.ab1 ............-..........-...........-..-..................... 108

1052-1_Microac_18_RARA-RARA_F_R.ab1 G....C......-.....T....-...........-.N--NT...........GNN.... 107

1062-1_Microac_19_RARA-RARA_F_R.ab1 ............-..........-...........-..-..................... 109

1066-1_Squamous_26_RARA-RARA_F_R.ab1 ............-..........-...........-..-..................... 110

1066-2_Squamous_27_RARA-RARA_F_R.ab1 ............-..........-...........-..-..................... 107

1139-1_EMT_30_RARA-RARA_F_R.ab1 N.NNNNNNNNNNTN..N.NC.N--..NN..NNN..C.TATNN.N....NTNT..--.TT. 109

1356-2_EMT_23_RARA-RARA_F_R.ab1 ............-..........-...........A.--..................... 108

1445-1_Squamous_28_RARA-RARA_F_R.ab1 ............-..........-...........A.--..................... 108

1576-1_Microac_20_RARA-RARA_F_R.ab1 G...........-N....T....-...........--.TN.................... 108

Consensus CCTCACCATCGMCGACCAGATCACCCTCCTCAAGGCTGCCTGCCTGGATATC-CTGATTC 169

222-1_EMT_21_RARA-RARA_F_R.ab1 ....T.....TC.C...CN..............N..................-....N.. 167

525-1_Squamous_24_RARA-RARA_F_R.ab1 ...........A........................................-....... 168

598-1_Squamous_25_RARA-RARA_F_R.ab1 ...........A........................................-....... 168

642-1_EMT_29_RARA-RARA_F_R.ab1 ...........C........................................-....... 169

812-1_EMT_22_RARA-RARA_F_R.ab1 ...........C........................................-....... 168

854-2_Microac_16_RARA-RARA_F_R.ab1 ...........A........................................-....... 169

864-1_Microac_17_RARA-RARA_F_R.ab1 ...........A........................................-....... 167

1052-1_Microac_18_RARA-RARA_F_R.ab1 ....T.....TC.C...................N..................-....... 166

1062-1_Microac_19_RARA-RARA_F_R.ab1 ...........A........................................-....... 168

1066-1_Squamous_26_RARA-RARA_F_R.ab1 ...........C........................................-....... 169

1066-2_Squamous_27_RARA-RARA_F_R.ab1 ...........C........................................-....... 166

1139-1_EMT_30_RARA-RARA_F_R.ab1 ...N...C..NC.C.A..A.N.N...C..A.TC..G..N.CNG......CNNAAA...C. 169

1356-2_EMT_23_RARA-RARA_F_R.ab1 ...........A........................................-....... 167

1445-1_Squamous_28_RARA-RARA_F_R.ab1 ...........A........................................-....... 167

1576-1_Microac_20_RARA-RARA_F_R.ab1 ..........TC.C......................................-....... 167

Consensus TGCGAATCTGCACGCGGTACACGCCTGAG-CAAGACACAAT-GACC-TTCTCAGATGGAC 226

222-1_EMT_21_RARA-RARA_F_R.ab1 .........NATAAAT.N....AAT.AT.N...C.T..NN.-....-.....TT...... 225

525-1_Squamous_24_RARA-RARA_F_R.ab1 .............................-...........-....-............. 225

598-1_Squamous_25_RARA-RARA_F_R.ab1 .............................-...........-....-............. 225

642-1_EMT_29_RARA-RARA_F_R.ab1 .............................-...........-....-............. 226

812-1_EMT_22_RARA-RARA_F_R.ab1 .............................-...........-....-............. 225

854-2_Microac_16_RARA-RARA_F_R.ab1 .............................-...........-....-............. 226

864-1_Microac_17_RARA-RARA_F_R.ab1 .............................-...........-....-............. 224

1052-1_Microac_18_RARA-RARA_F_R.ab1 ...............N.N...........-...C....NN.-....-.....TT...... 223

1062-1_Microac_19_RARA-RARA_F_R.ab1 .............................-...........-....-............. 225

1066-1_Squamous_26_RARA-RARA_F_R.ab1 .............................-...........-....-............. 226

1066-2_Squamous_27_RARA-RARA_F_R.ab1 .............................-...........-....-............. 223

1139-1_EMT_30_RARA-RARA_F_R.ab1 G.A...-.A..C..G...N.-.TT....---N.N.N.A.N.A.N..N..T..--N.N... 222

1356-2_EMT_23_RARA-RARA_F_R.ab1 .............................-...........-....-............. 224

1445-1_Squamous_28_RARA-RARA_F_R.ab1 .............................-...........-....-............. 224

1576-1_Microac_20_RARA-RARA_F_R.ab1 .................N...........-...C....N..-....-.....TT...... 224

Consensus TGACCCTGAACCGGACTCAGATGCACAAAGCTGAGWTTTGGAAWGACWTTGGGARCAACW 286

222-1_EMT_21_RARA-RARA_F_R.ab1 .........TG..A....NC...A.AN........T.......A...T......G...NT 285

525-1_Squamous_24_RARA-RARA_F_R.ab1 .............................------------------------------- 285

598-1_Squamous_25_RARA-RARA_F_R.ab1 .............................------------------------------- 285

642-1_EMT_29_RARA-RARA_F_R.ab1 ...............NNN-N.........------------------------------- 285

812-1_EMT_22_RARA-RARA_F_R.ab1 .............................------------------------------- 285

854-2_Microac_16_RARA-RARA_F_R.ab1 .............................------------------------------- 286

864-1_Microac_17_RARA-RARA_F_R.ab1 .............................------------------------------- 284

1052-1_Microac_18_RARA-RARA_F_R.ab1 .........TG..A....CC...A...........T.......A...T......G....N 283

1062-1_Microac_19_RARA-RARA_F_R.ab1 .............................------------------------------- 285

1066-1_Squamous_26_RARA-RARA_F_R.ab1 .............................------------------------------- 286

1066-2_Squamous_27_RARA-RARA_F_R.ab1 .............................------------------------------- 283

1139-1_EMT_30_RARA-RARA_F_R.ab1 .NN..TG.....T.T..TNN.....A..T.N----------------------------- 282

1356-2_EMT_23_RARA-RARA_F_R.ab1 .............................------------------------------- 284

1445-1_Squamous_28_RARA-RARA_F_R.ab1 .............................------------------------------- 284

1576-1_Microac_20_RARA-RARA_F_R.ab1 .........TG..A....CC..............NT.......A...T......G....A 284

Consensus TTGGGRSMA 295

222-1_EMT_21_RARA-RARA_F_R.ab1 .G..AGCA- 293

525-1_Squamous_24_RARA-RARA_F_R.ab1 --------- 254

598-1_Squamous_25_RARA-RARA_F_R.ab1 --------- 254

642-1_EMT_29_RARA-RARA_F_R.ab1 --------- 254

812-1_EMT_22_RARA-RARA_F_R.ab1 --------- 254

854-2_Microac_16_RARA-RARA_F_R.ab1 --------- 255

864-1_Microac_17_RARA-RARA_F_R.ab1 --------- 253

1052-1_Microac_18_RARA-RARA_F_R.ab1 .....AGC. 292

1062-1_Microac_19_RARA-RARA_F_R.ab1 --------- 254

1066-1_Squamous_26_RARA-RARA_F_R.ab1 --------- 255

1066-2_Squamous_27_RARA-RARA_F_R.ab1 --------- 252

1139-1_EMT_30_RARA-RARA_F_R.ab1 --------- 253

1356-2_EMT_23_RARA-RARA_F_R.ab1 --------- 253

1445-1_Squamous_28_RARA-RARA_F_R.ab1 --------- 253

1576-1_Microac_20_RARA-RARA_F_R.ab1 .....NGN- 292

 
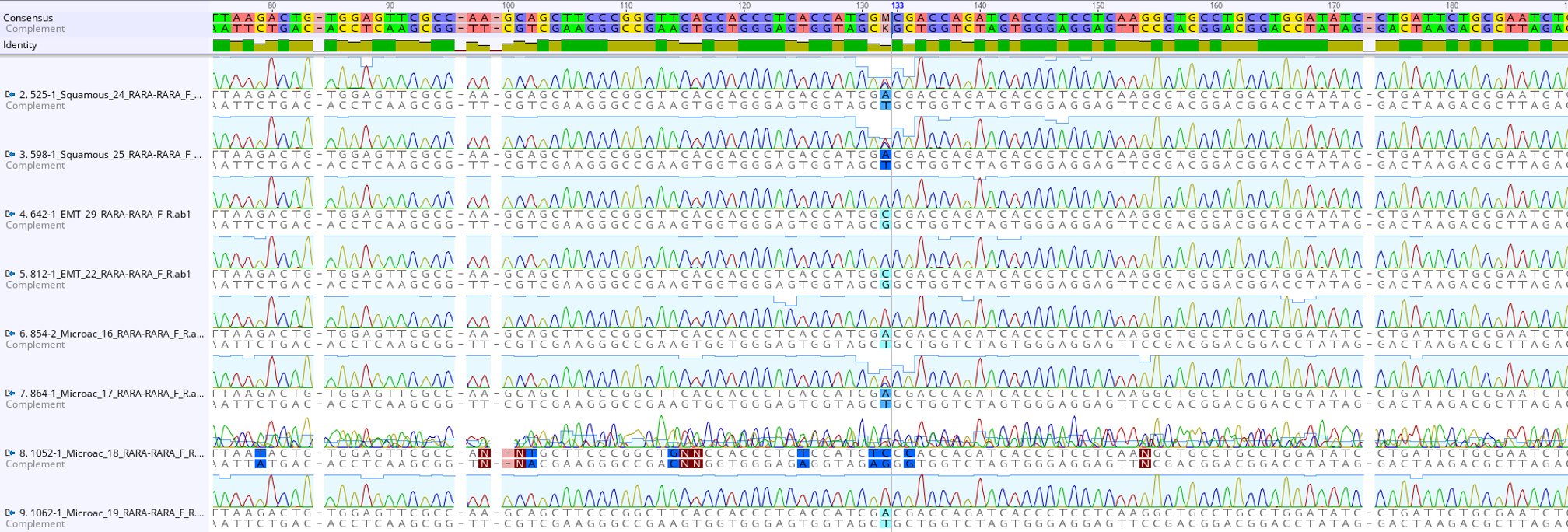

Supplement: Supplementary file 2 — Additional file 2: Aligned sanger sequencing results of the same 5 microacinar, 5 squamous, and 5 EMT tumors over RARα. Shows a conserved C to A mutation present at position 132 in the electropherogram. These mutations occur in the same tumors as the KIT C to A mutations (525-1, 598-1, 864-1, 1052-1, 1356-2, 1445-1, and 1576-1). [file 13058_2023_1723_MOESM2_ESM.docx]
