# Supplementary figures and images for: Integrative multi-omic sequencing reveals the MMTV-Myc mouse model mimics human breast cancer heterogeneity
Source: Breast Cancer Res. 2023 Oct 7;25:120. doi: 10.1186/s13058-023-01723-3 (PMC10559619; doi:10.1186/s13058-023-01723-3)

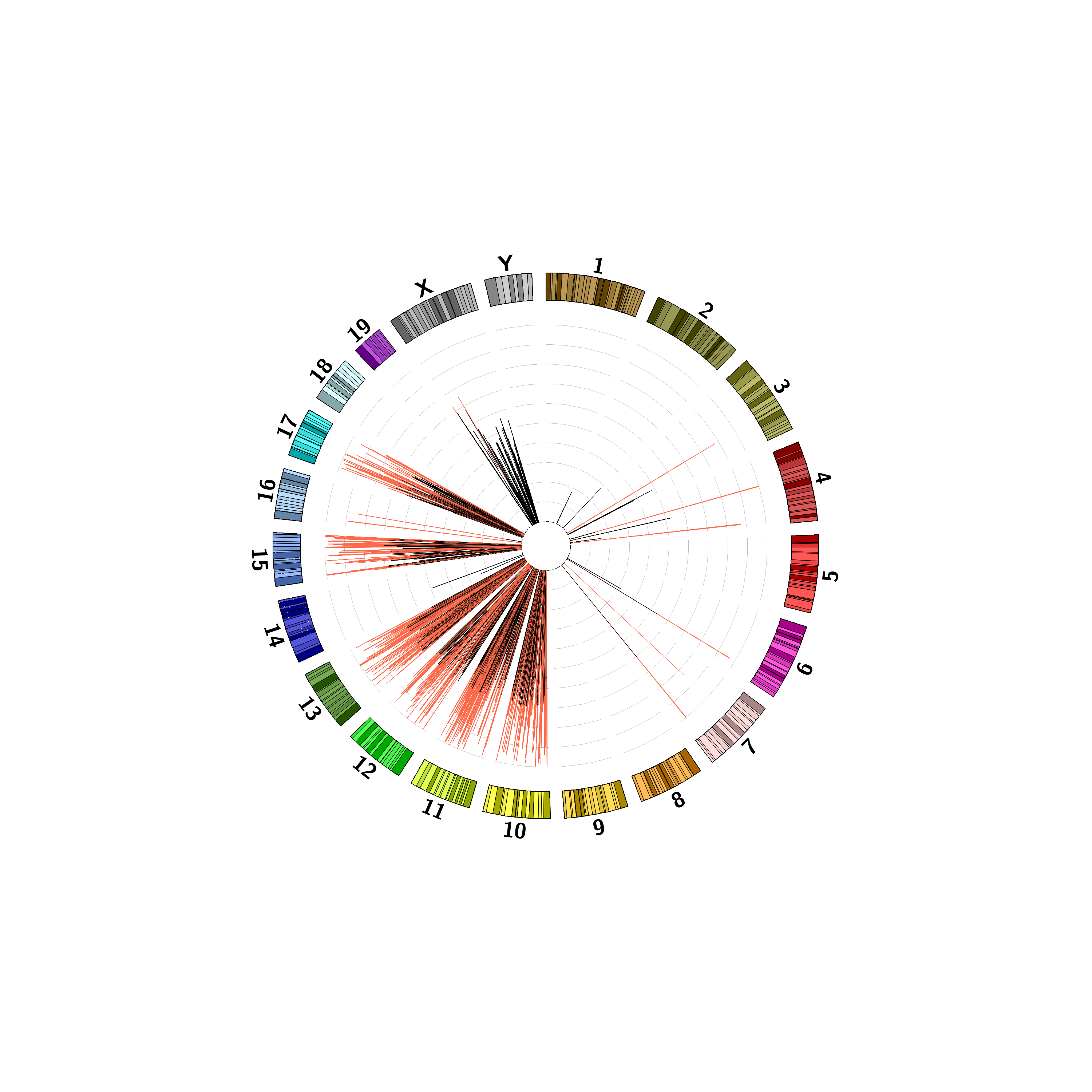

Supplement: Supplementary file 5 — Additional file 5: Fig. S1. Circos plot of Pearson correlation coefficient values for integer copy number and gene expression float value for the EMT histological subtype alone. Red indicates that the correlation value is above 0.7, while being colored black means the correlation value is below 0.7. [file 13058_2023_1723_MOESM5_ESM.png]

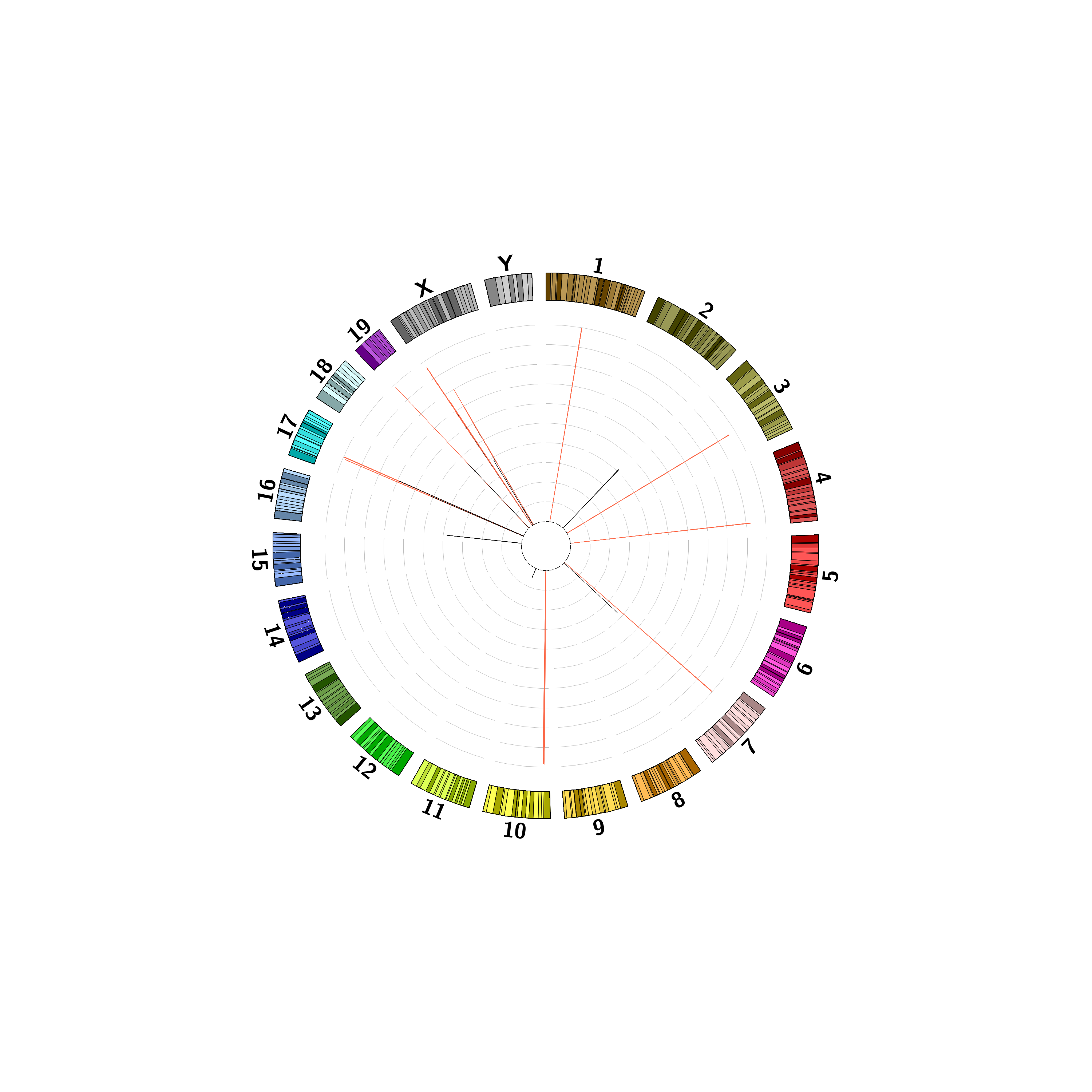

Supplement: Supplementary file 6 — Additional file 6: Fig. S2. Circos plot of Pearson correlation coefficient values for integer copy number and gene expression float value for the squamous histological subtype alone. Red indicates that the correlation value is above 0.7, while being colored black means the correlation value is below 0.7. [file 13058_2023_1723_MOESM6_ESM.png]

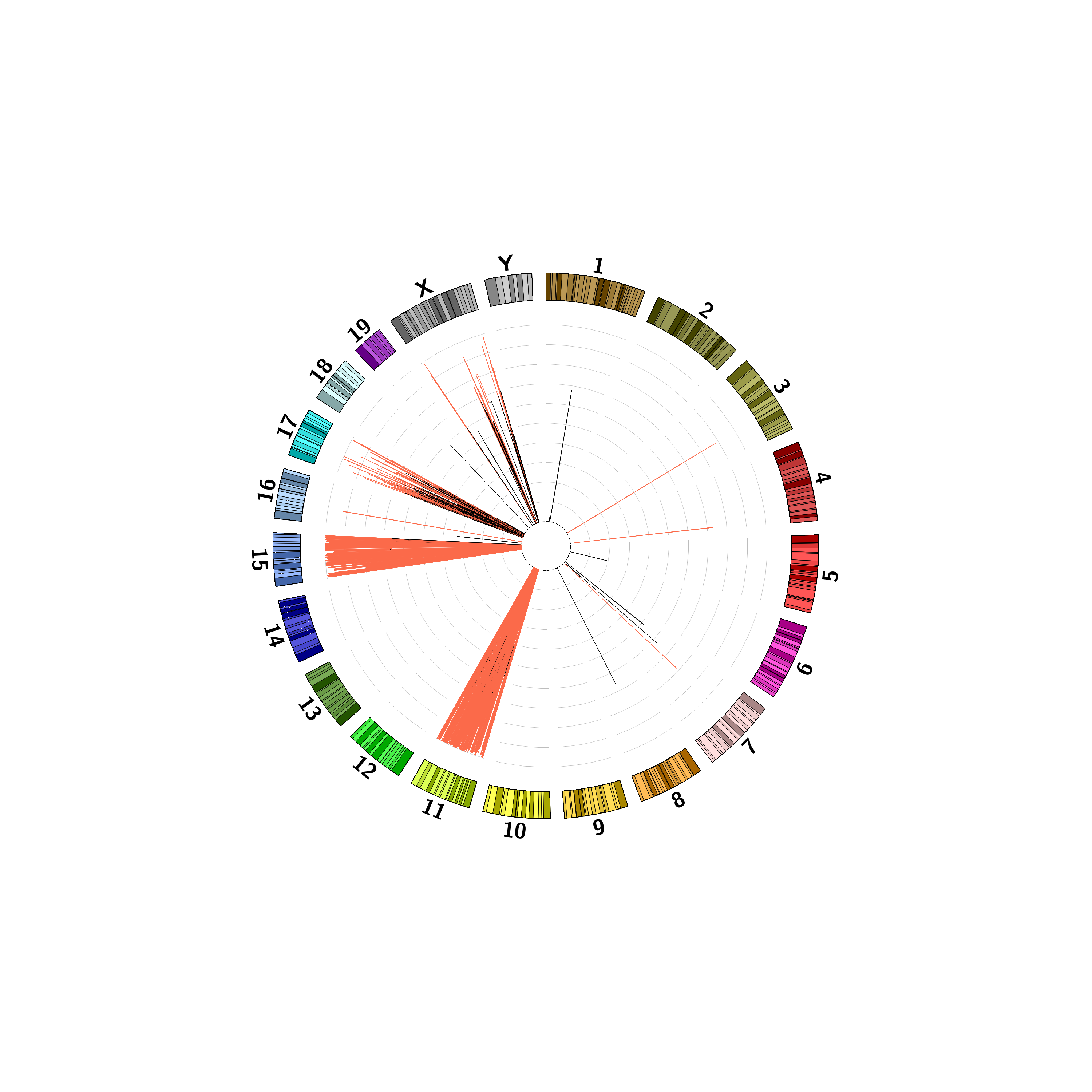

Supplement: Supplementary file 7 — Additional file 7: Fig. S3. Circos plot of Pearson correlation coefficient values for integer copy number and gene expression float value for the microacinar histological subtype alone. Red indicates that the correlation value is above 0.7, while being colored black means the correlation value is below 0.7. [file 13058_2023_1723_MOESM7_ESM.png]

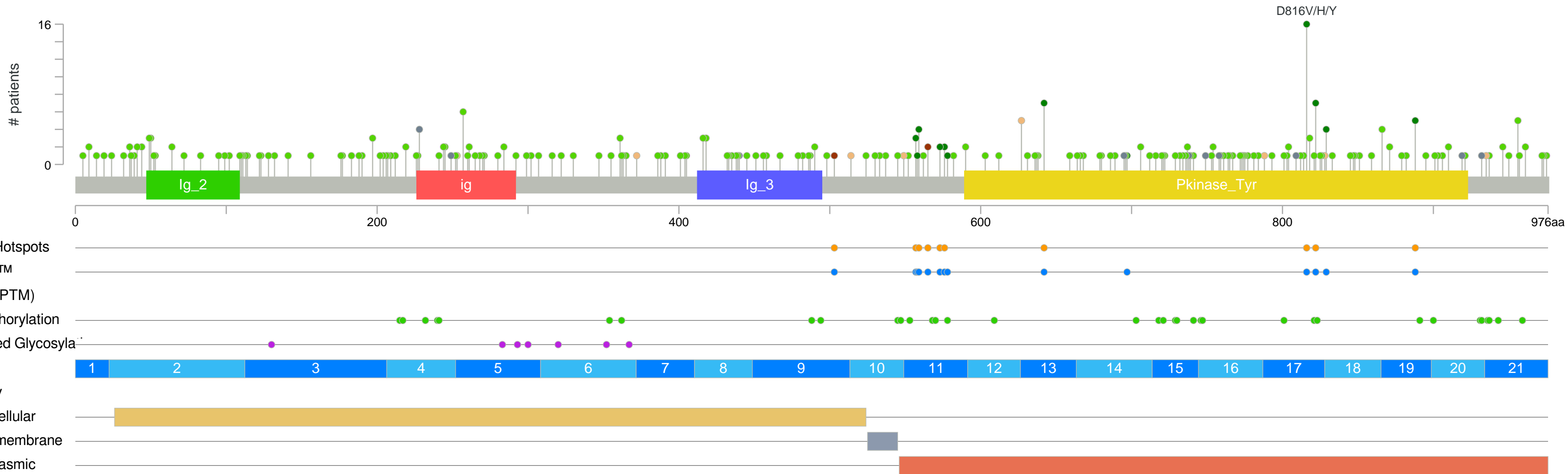

Supplement: Supplementary file 8 — Additional file 8: Fig. S4. Lollipop plot for human KIT (isoform 1) protein obtained from cBioPortal. Patient samples are from the TCGA PanCancer Atlas. The lollipops represent different mutations, with light green representing missense mutations of unknown pathology. Dark green mutations are confirmed pathologic missense mutations. Gray mutations represent truncating mutations of unknown pathology. Beige mutations represent splice variants of unknown pathology. Brown mutations represent inframe deletions or insertions of unknown pathology. Dark brown mutations represent confirmed pathologic inframe deletions or insertions. The annotation tracks below the plot correspond to the labels designated on the left. Each dot on the annotation track maps back to that location on the protein. [file 13058_2023_1723_MOESM8_ESM.pdf]

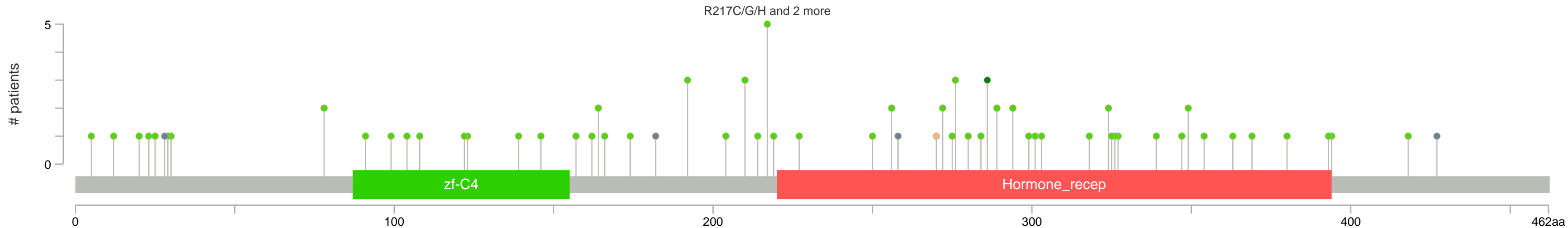

Cancer Hotspots

OncoKB™

PTM (dbPTM)

Phosphorylation

Sumoylation

Exon

Topology

2

3

4

5

6

7

8

9

Supplement: Supplementary file 9 — Additional file 9: Fig. S5. Lollipop plot for human RARA (isoform 1) protein obtained from cBioPortal. Patient samples are from the TCGA PanCancer Atlas. The lollipops represent different mutations, with light green representing missense mutations of unknown pathology. Dark green mutations are confirmed pathologic missense mutations. Gray mutations represent truncating mutations of unknown pathology. Beige mutations represent splice variants of unknown pathology. Brown mutations represent inframe deletions or insertions of unknown pathology. The annotation tracks below the plot correspond to the labels designated on the left. Each dot on the annotation track maps back to that location on the protein. [file 13058_2023_1723_MOESM9_ESM.pdf]

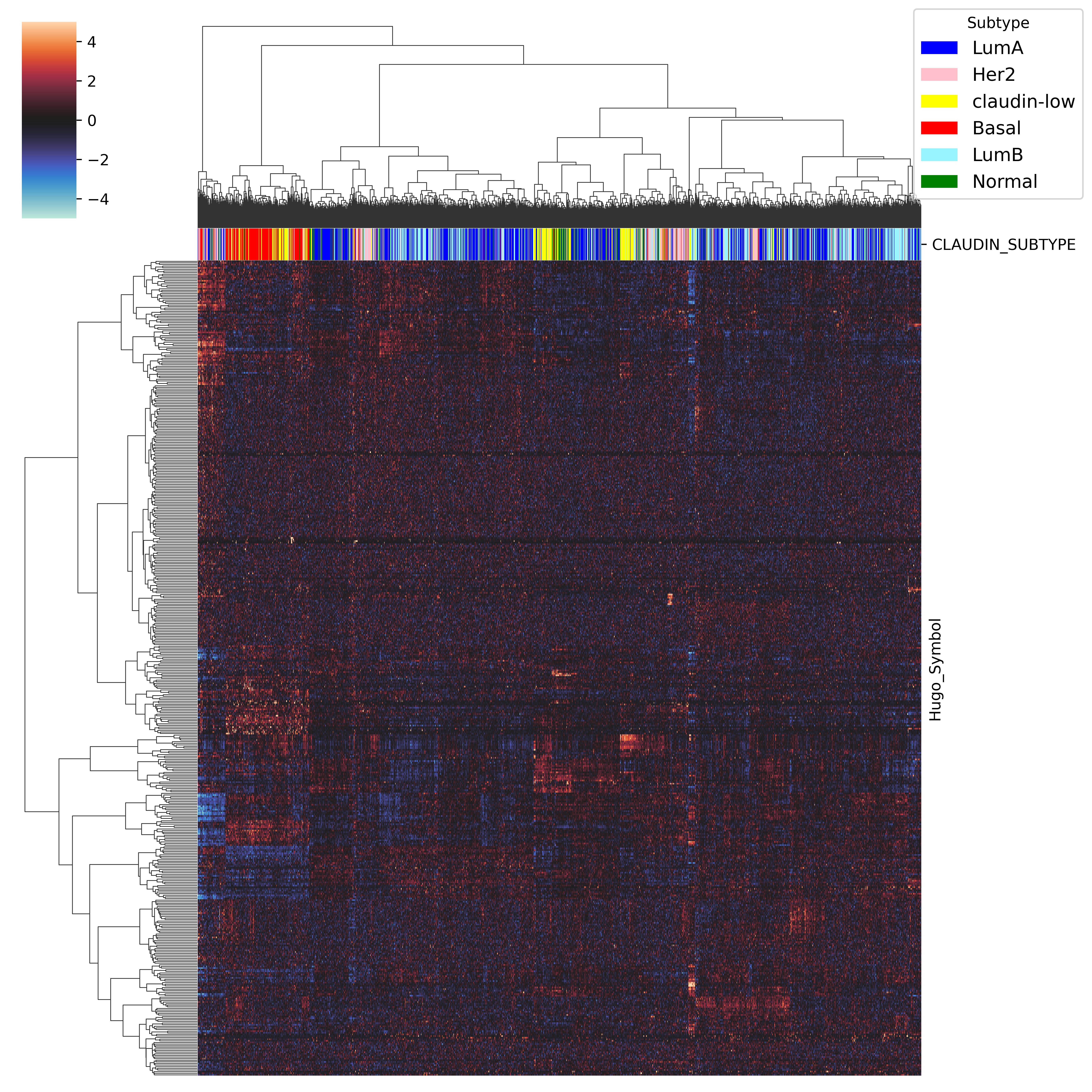

Supplement: Supplementary file 11 — Additional file 11: Fig. S7. METABRIC gene expression data with 446 random genes selected as the feature set and then undergoing unsupervised hierarchical clustering using Seaborn’s clustermap function within Python. This is the first random instantiation. [file 13058_2023_1723_MOESM11_ESM.png]

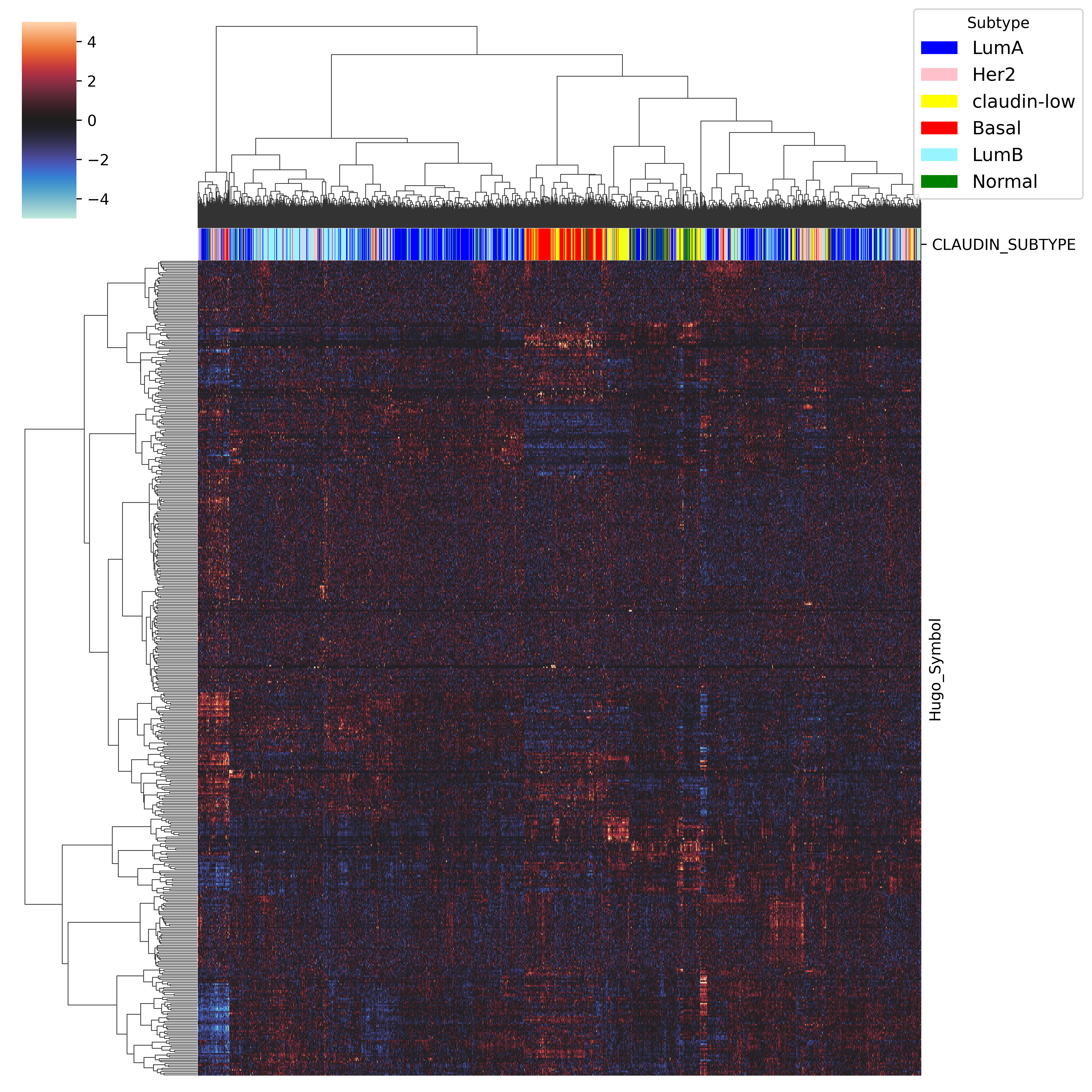

Supplement: Supplementary file 12 — Additional file 12: Fig. S8. METABRIC gene expression data with 446 random genes selected as the feature set and then undergoing unsupervised hierarchical clustering using Seaborn’s clustermap function within Python. This is the second random instantiation. [file 13058_2023_1723_MOESM12_ESM.png]

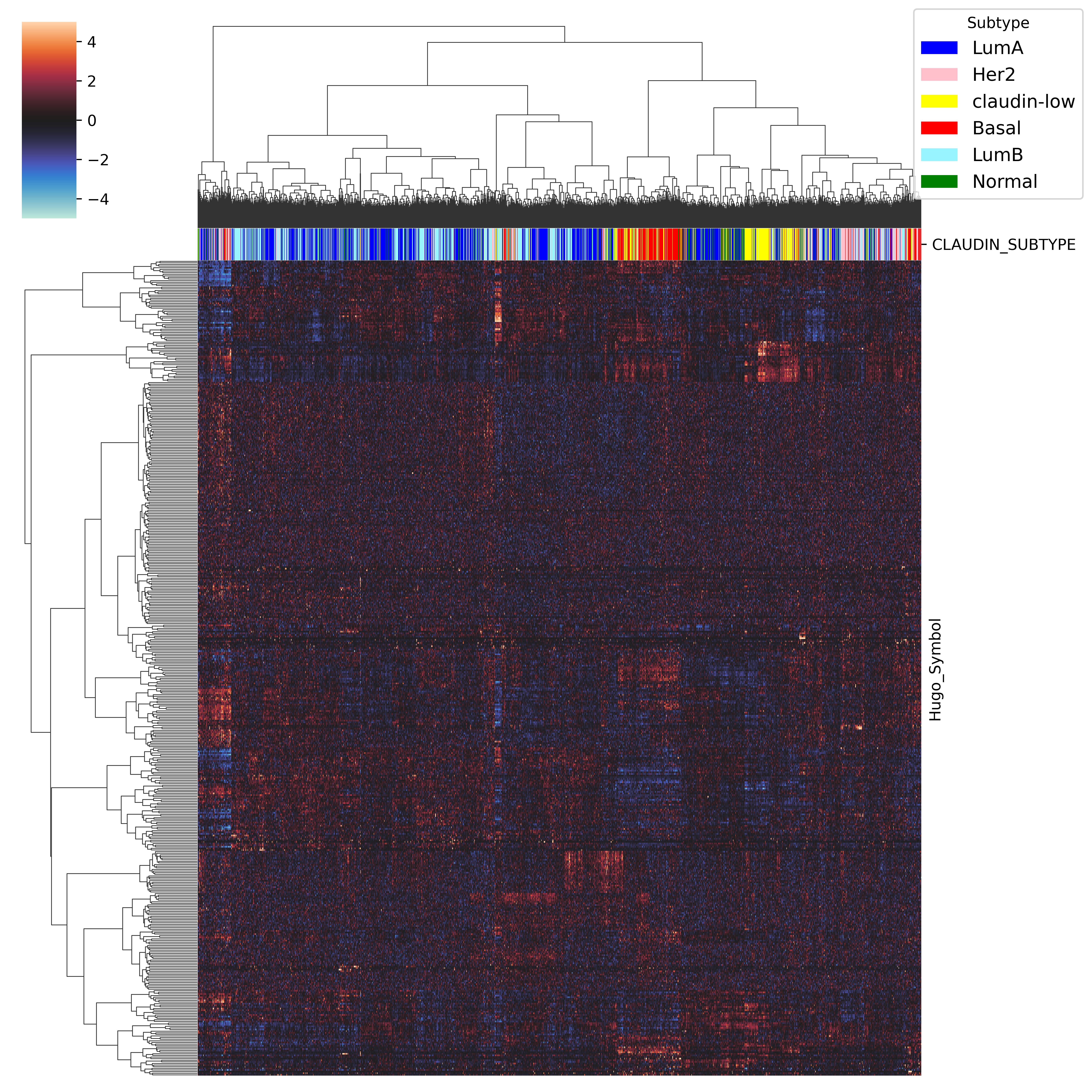

Supplement: Supplementary file 13 — Additional file 13: Fig. S9. METABRIC gene expression data with 446 random genes selected as the feature set and then undergoing unsupervised hierarchical clustering using Seaborn’s clustermap function within Python. This is the third random instantiation. [file 13058_2023_1723_MOESM13_ESM.png]

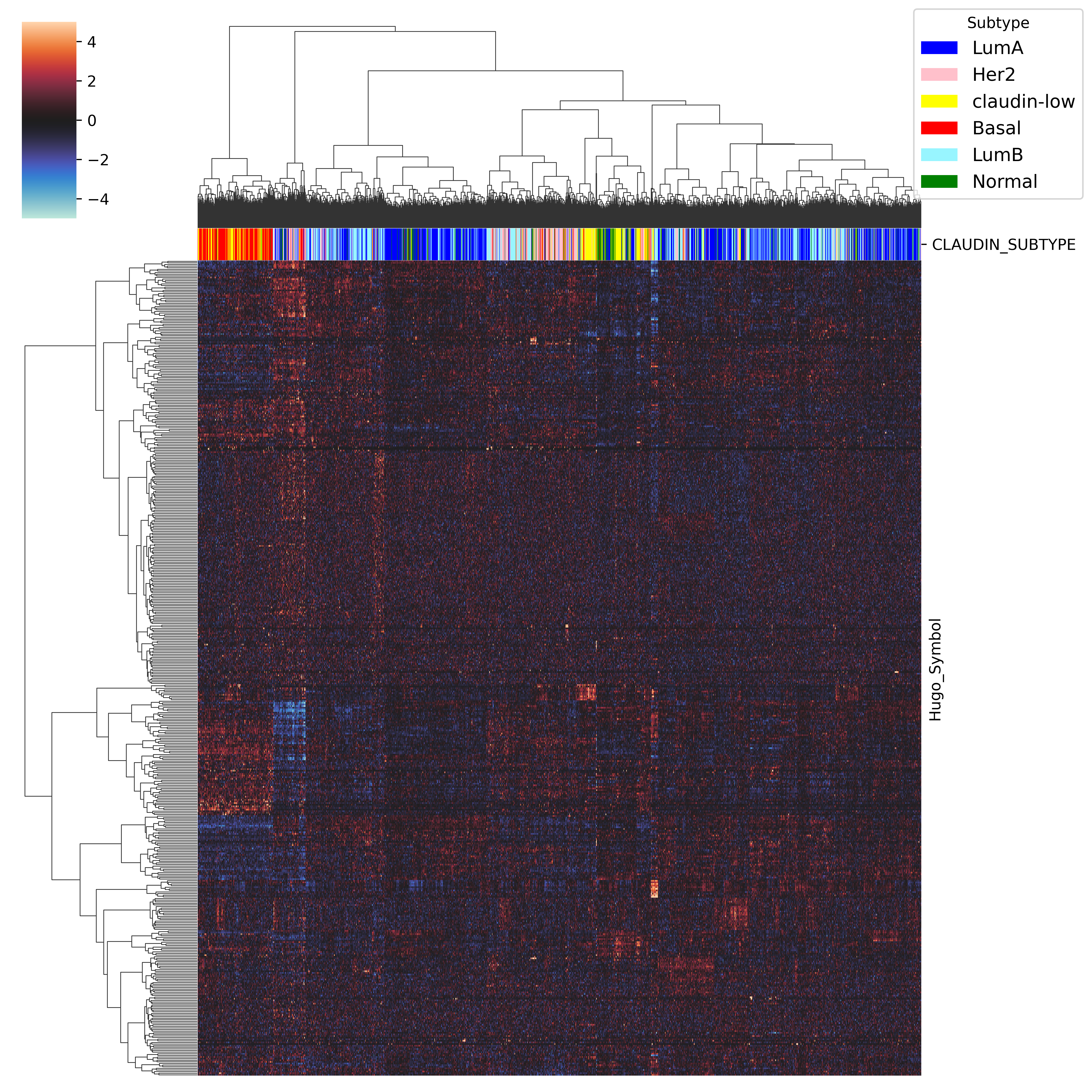

Supplement: Supplementary file 14 — Additional file 14: Fig. S10. METABRIC gene expression data with 446 random genes selected as the feature set and then undergoing unsupervised hierarchical clustering using Seaborn’s clustermap function within Python. This is the fourth random instantiation. [file 13058_2023_1723_MOESM14_ESM.png]

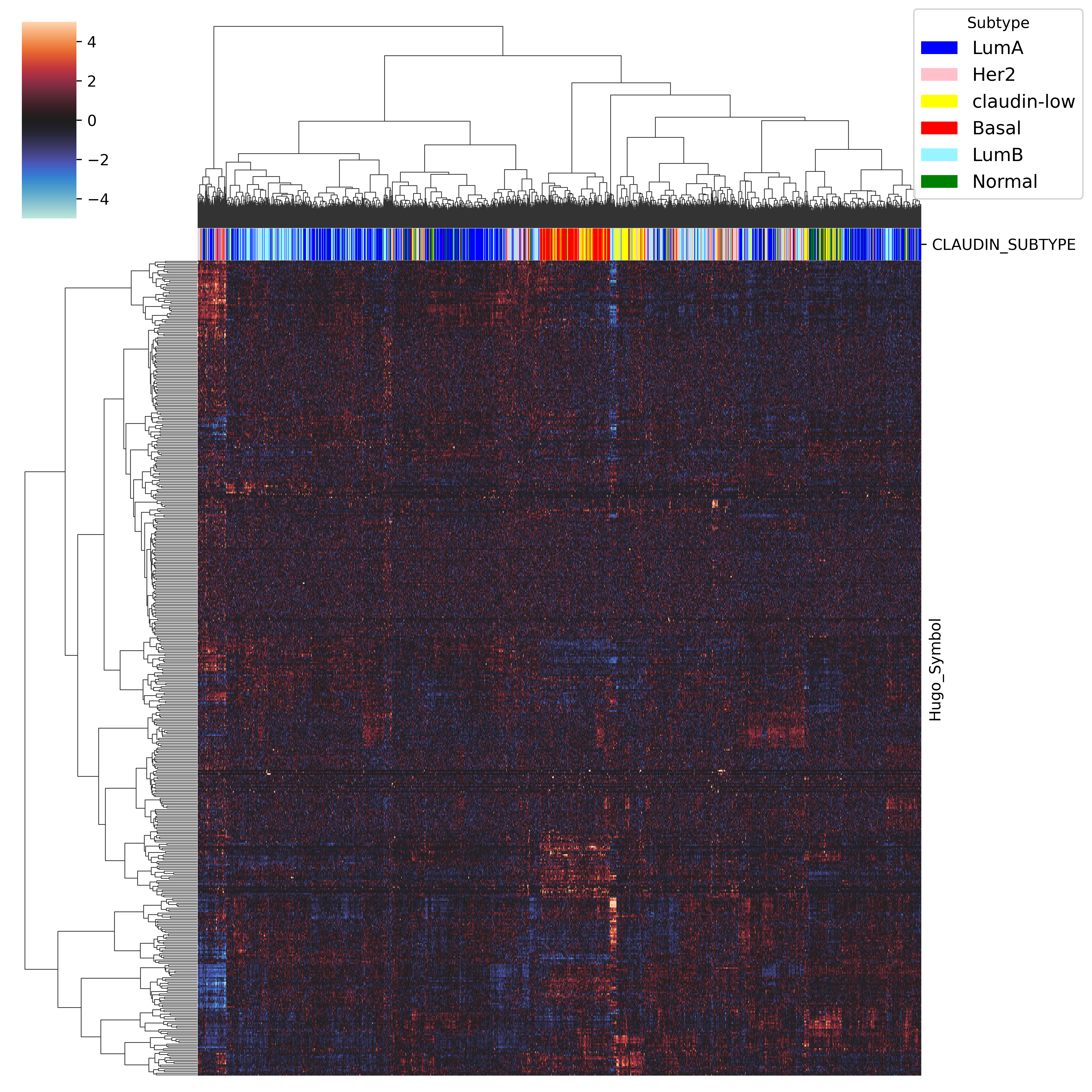

Supplement: Supplementary file 15 — Additional file 15: Fig. S11. METABRIC gene expression data with 446 random genes selected as the feature set and then undergoing unsupervised hierarchical clustering using Seaborn’s clustermap function within Python. This is the fifth random instantiation. [file 13058_2023_1723_MOESM15_ESM.png]

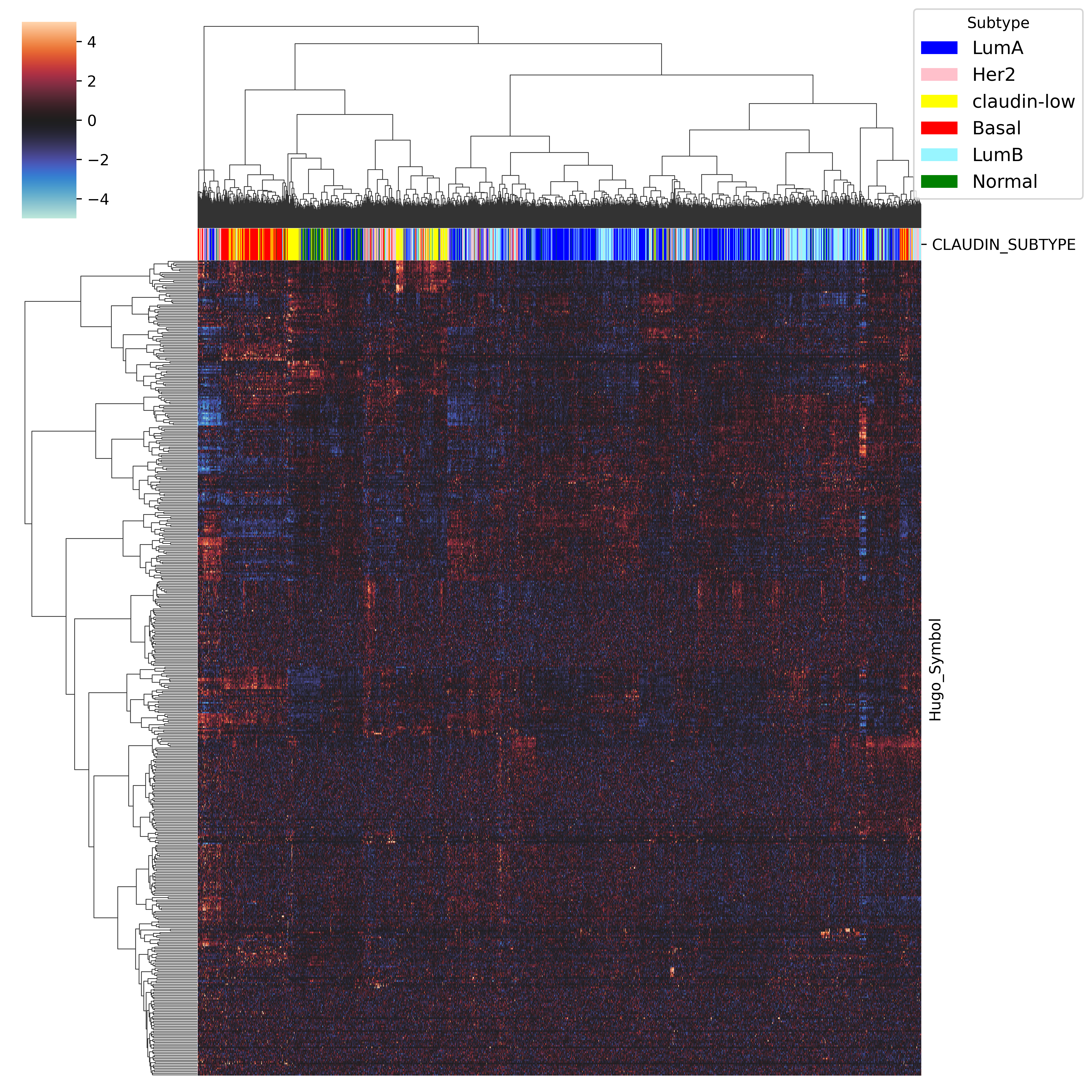

Supplement: Supplementary file 16 — Additional file 16: Fig. S12. METABRIC gene expression data with 446 random genes selected as the feature set and then undergoing unsupervised hierarchical clustering using Seaborn’s clustermap function within Python. This is the sixth random instantiation. [file 13058_2023_1723_MOESM16_ESM.png]

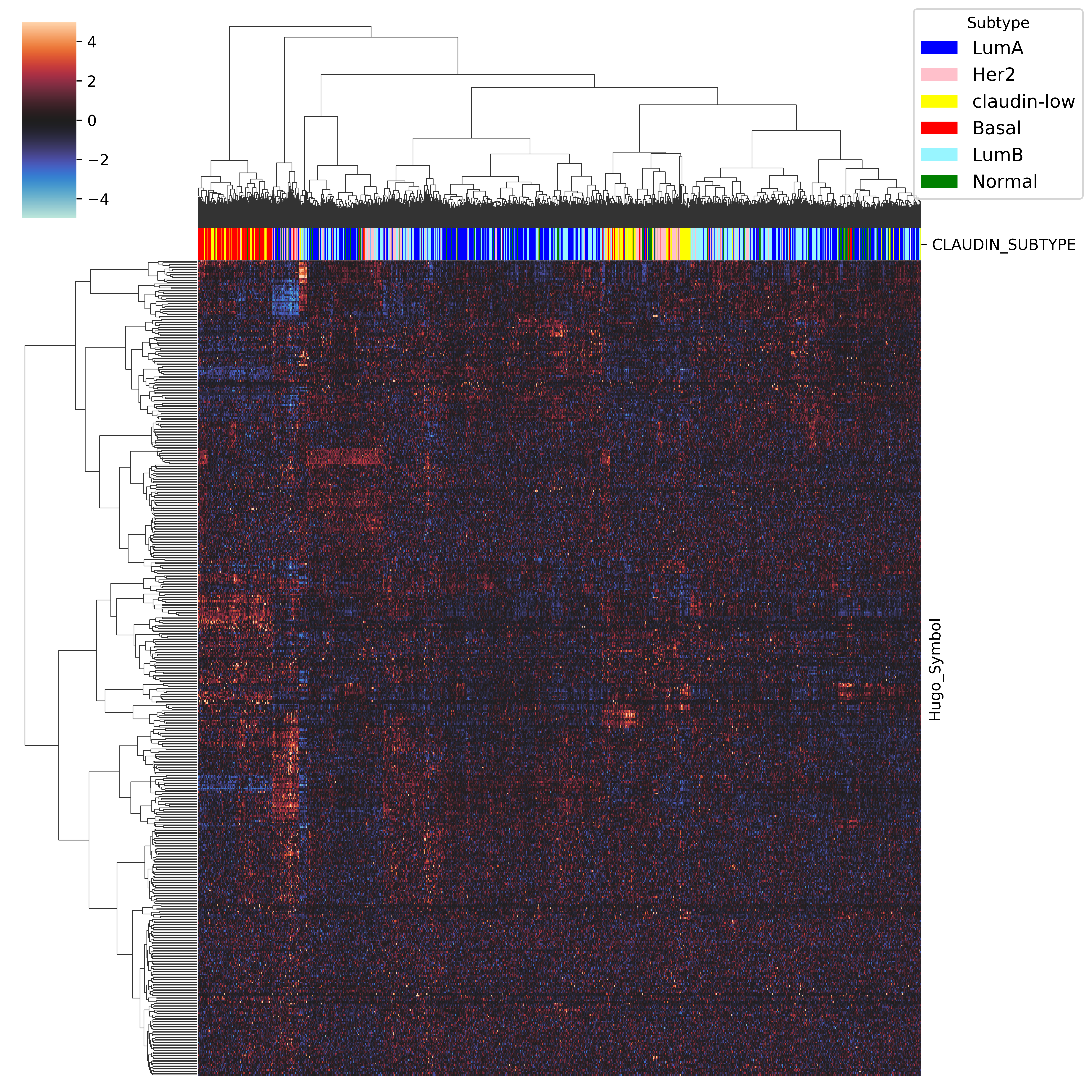

Supplement: Supplementary file 17 — Additional file 17: Fig. S13. METABRIC gene expression data with 446 random genes selected as the feature set and then undergoing unsupervised hierarchical clustering using Seaborn’s clustermap function within Python. This is the seventh random instantiation. [file 13058_2023_1723_MOESM17_ESM.png]

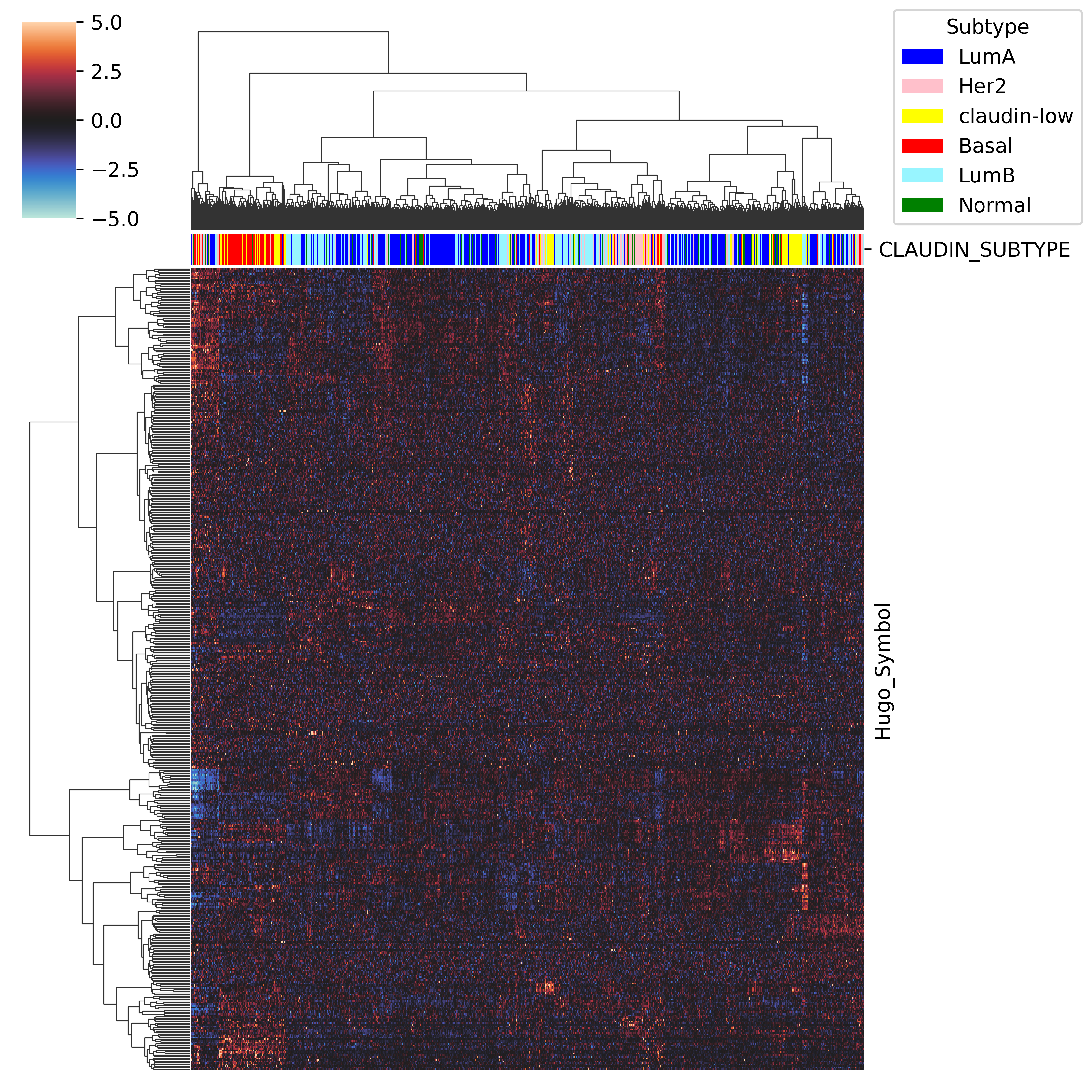

Supplement: Supplementary file 18 — Additional file 18: Fig. S14. METABRIC gene expression data with 446 random genes selected as the feature set and then undergoing unsupervised hierarchical clustering using Seaborn’s clustermap function within Python. This is the eighth random instantiation. [file 13058_2023_1723_MOESM18_ESM.png]

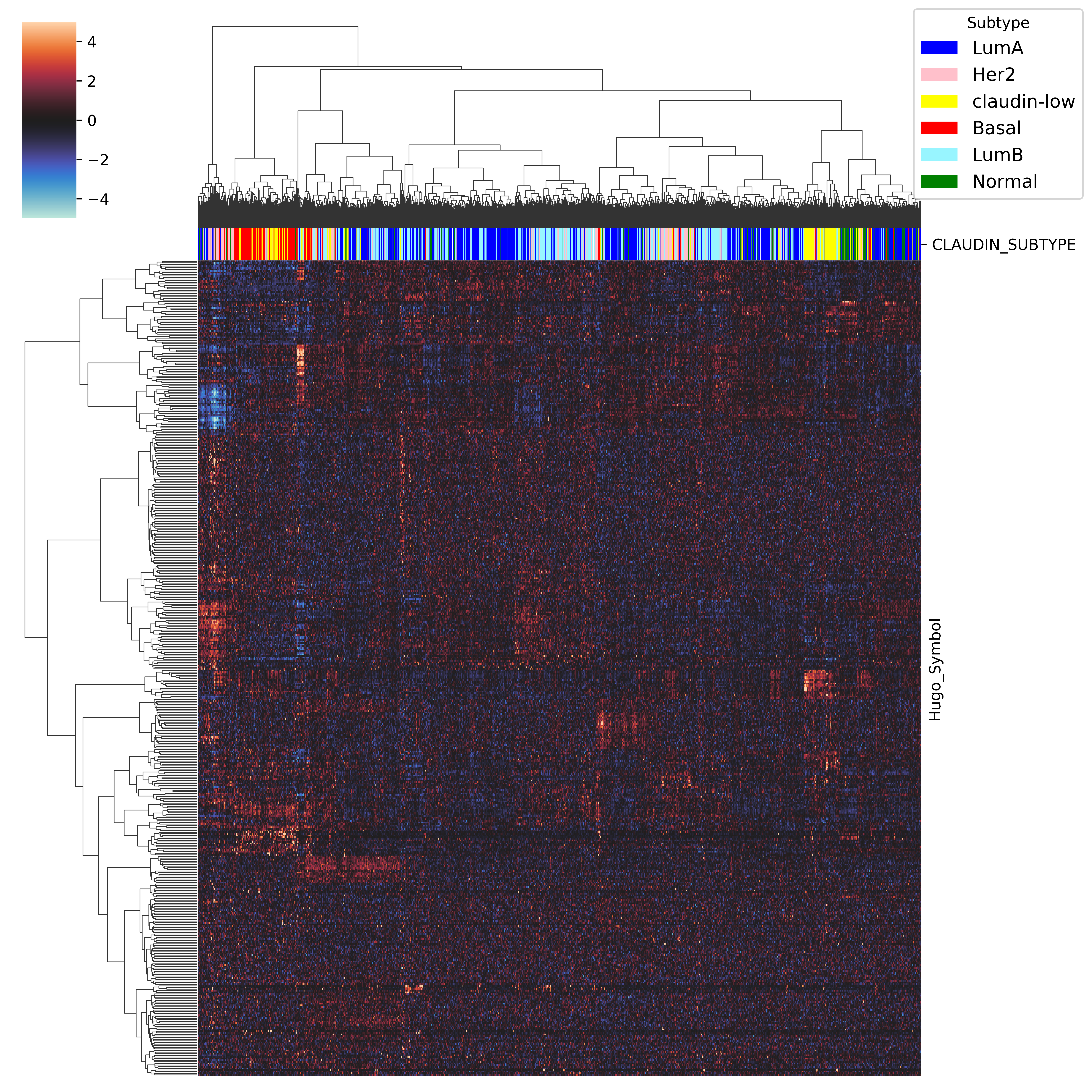

Supplement: Supplementary file 19 — Additional file 19: Fig. S15. METABRIC gene expression data with 446 random genes selected as the feature set and then undergoing unsupervised hierarchical clustering using Seaborn’s clustermap function within Python. This is the ninth random instantiation. [file 13058_2023_1723_MOESM19_ESM.png]

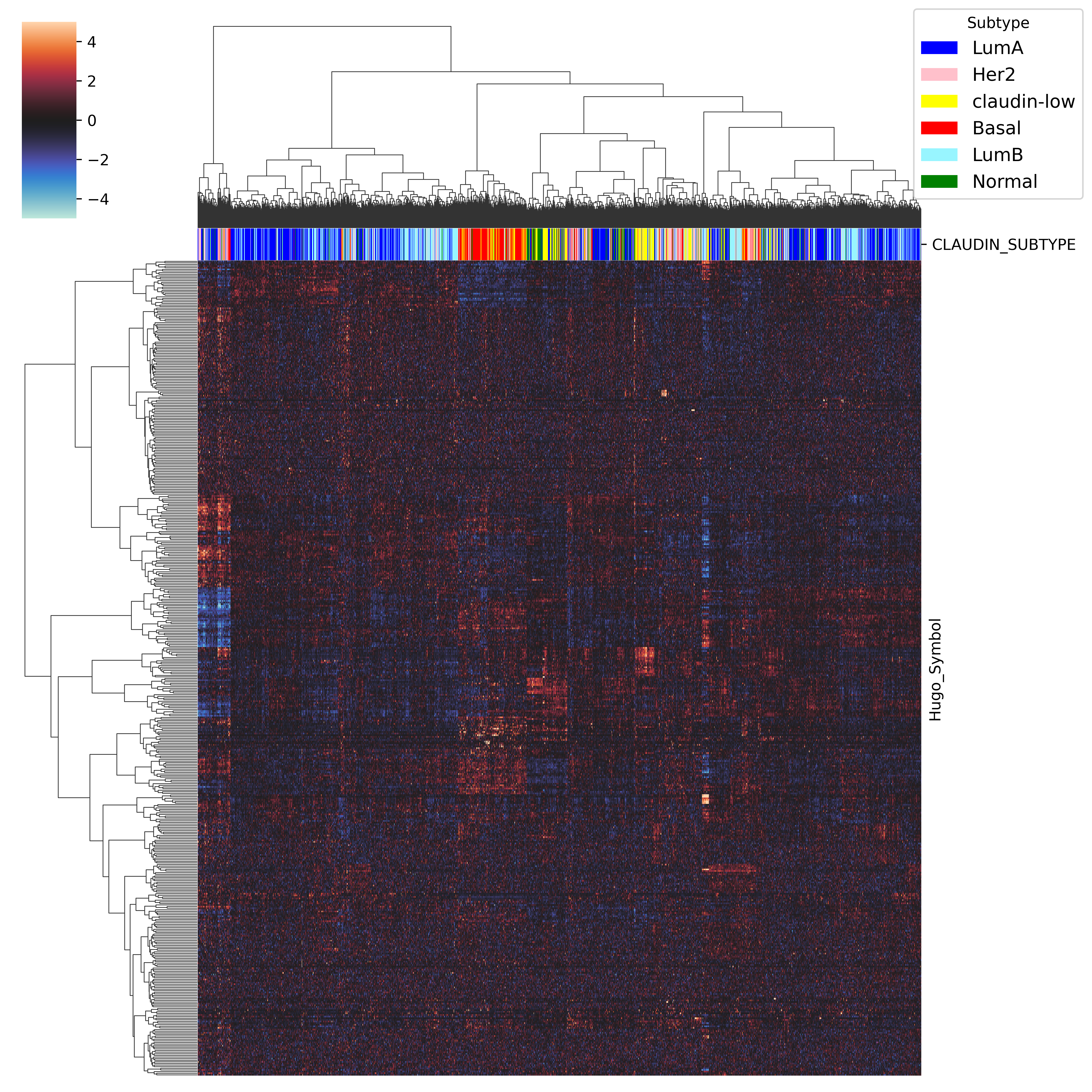

Supplement: Supplementary file 20 — Additional file 20: Fig. S16. METABRIC gene expression data with 446 random genes selected as the feature set and then undergoing unsupervised hierarchical clustering using Seaborn’s clustermap function within Python. This is the tenth random instantiation. [file 13058_2023_1723_MOESM20_ESM.png]

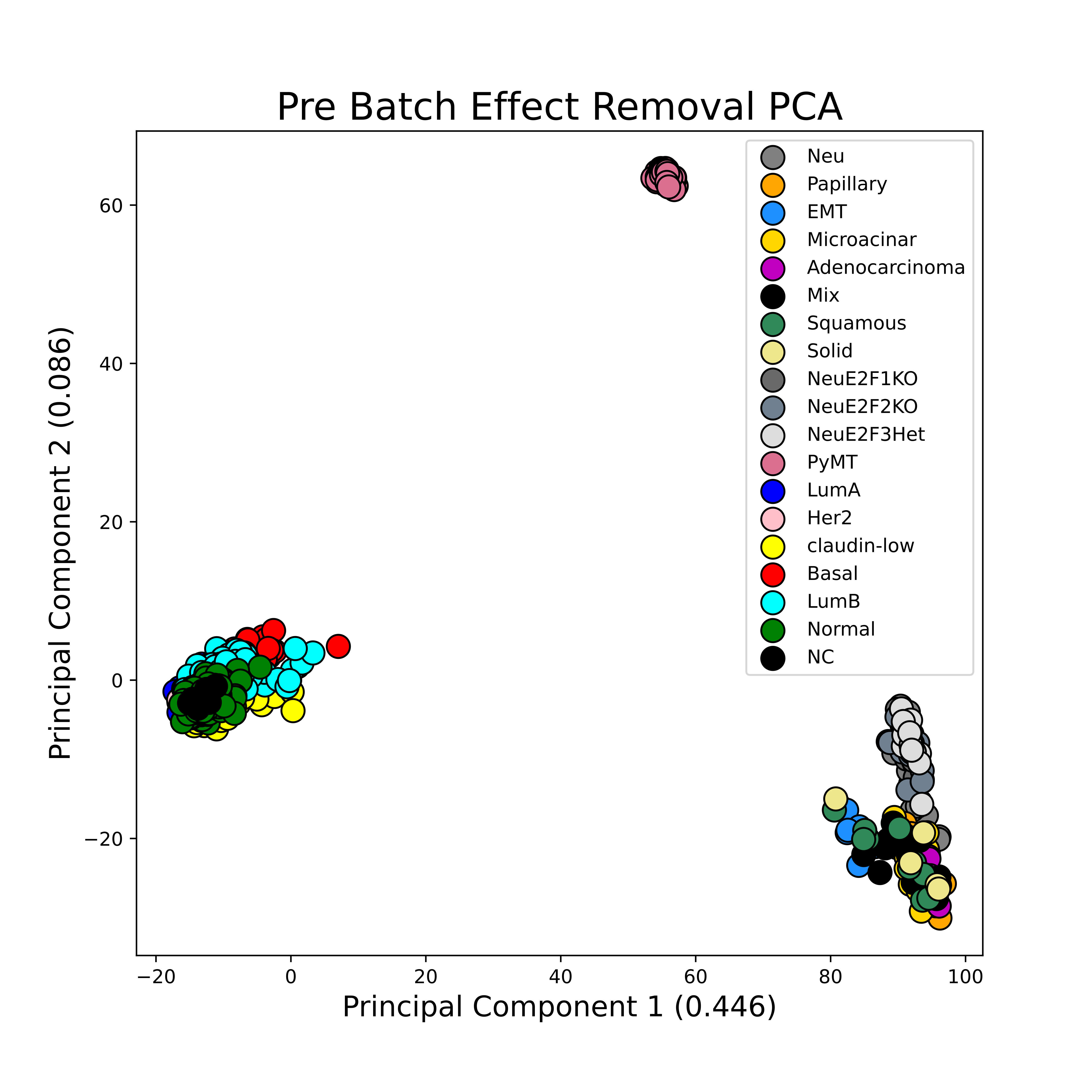

Supplement: Supplementary file 21 — Additional file 21: Fig. S17. PCA plot of Z-scaled data for METABRIC gene expression data, and also MMTV-Myc, MMTV-Neu, and MMTV-PyMT primary mammary mouse tumors and visualized using a matplotlib scatterplot in Python. The first principal component accounts for 44.6% of the variance within the dataset, with three distinct clusters that correspond to different sampling groups appearing. The second principal component accounts for 8.6% of variance within the dataset. [file 13058_2023_1723_MOESM21_ESM.png]

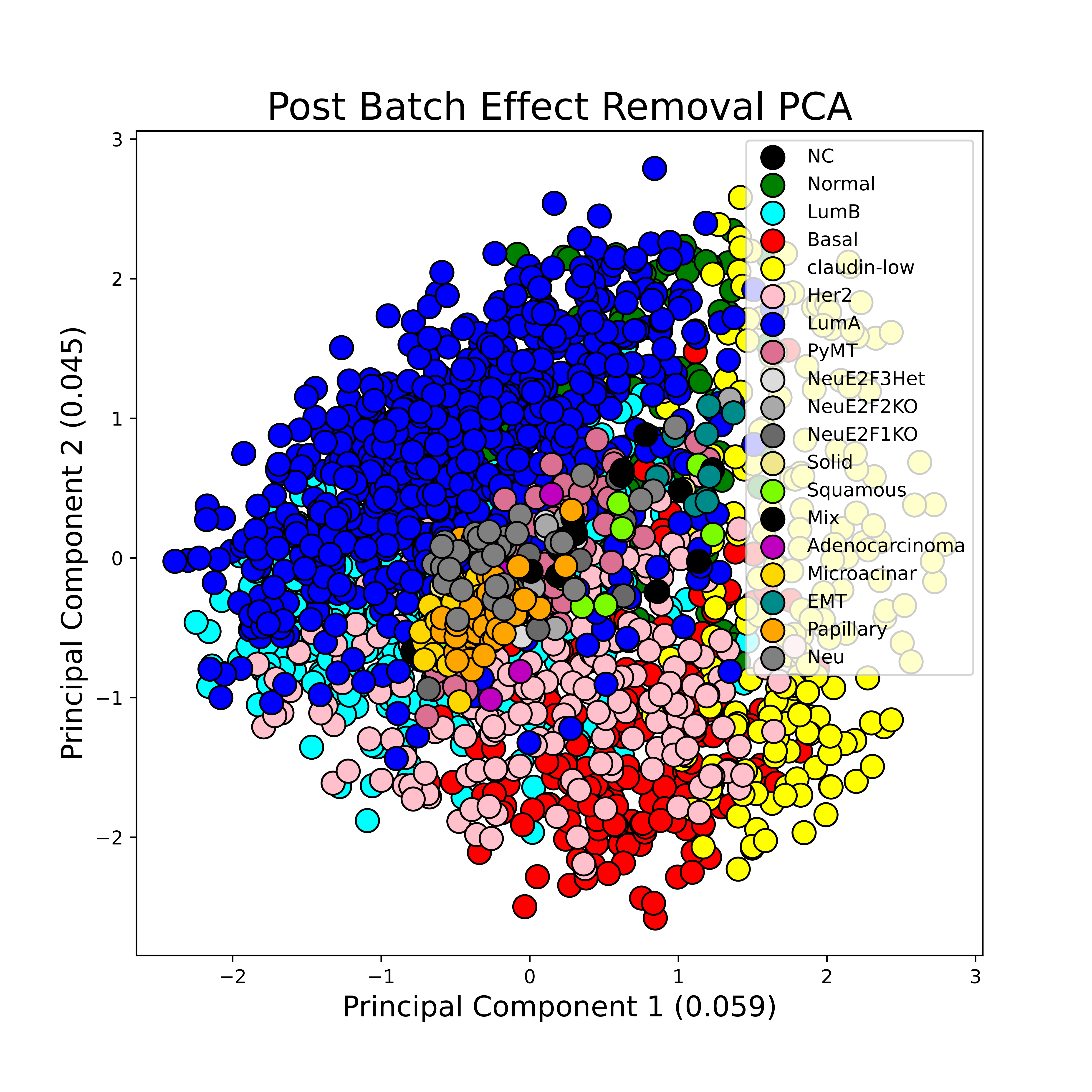

Supplement: Supplementary file 22 — Additional file 22: Fig. S18. PCA plot of Z-scaled and batch corrected METABRIC, MMTV-Myc, MMTV-Neu, and MMTV-PyMT primary mammary tumor gene expression data. Batch correction was performed using ComBat with settings defined in the main text methods section. Principal components are plotted using a scatterplot in matplotlib within Python. The first principal component in the batch corrected dataset accounts for 5.9% of variance, while the second principal component accounts for 4.5% of variance. [file 13058_2023_1723_MOESM22_ESM.png]

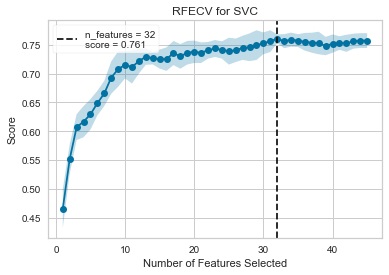

Supplement: Supplementary file 23 — Additional file 23: Fig. S19. Recursive feature elimination with 10-fold cross-validation (RFECV) using a support vector machine (SVM) classifier with radial basis function (RBF) kernel was performed on Z-scored and batch corrected METABRIC gene expression data—all mouse data was removed after batch correction and Z-scoring but before RFECV. Gene expression data was limited to the remaining PAM50 genes after combining human and mouse datasets, which was 45 genes. The vertical dashed line was placed at the optimal number of features, which is the number of features which give the highest average accuracy score over the 10 iterations the test is performed. The light blue shaded area represents one standard deviation away from the mean accuracy score. RFECV was performed using the Yellowbrick package in Python. [file 13058_2023_1723_MOESM23_ESM.jpg]

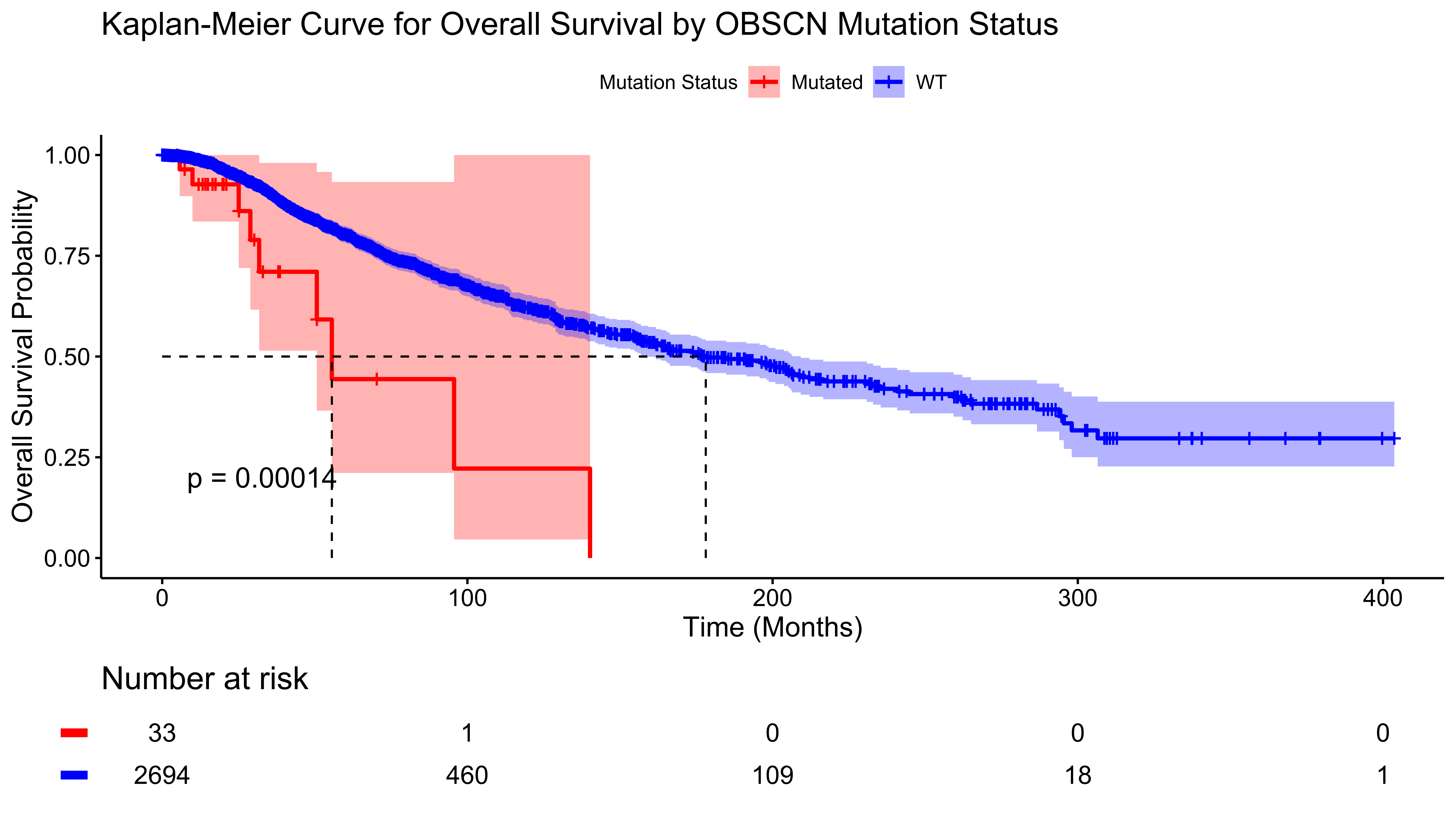

Supplement: Supplementary file 24 — Additional file 24: Fig. S20. Non-redundant TCGA breast cancer patient data for OBSCN mutation status underwent Kaplan-Meier analysis for overall survival with 95% confidence intervals shown in light blue for the unaltered population and light red for the OBSCN mutated population. KM plots were created using the survminer R package. All breast cancer datasets with mutation data available were analyzed with redundant patients removed from the dataset and the first event occurrence by date for each patient was kept. [file 13058_2023_1723_MOESM24_ESM.png]

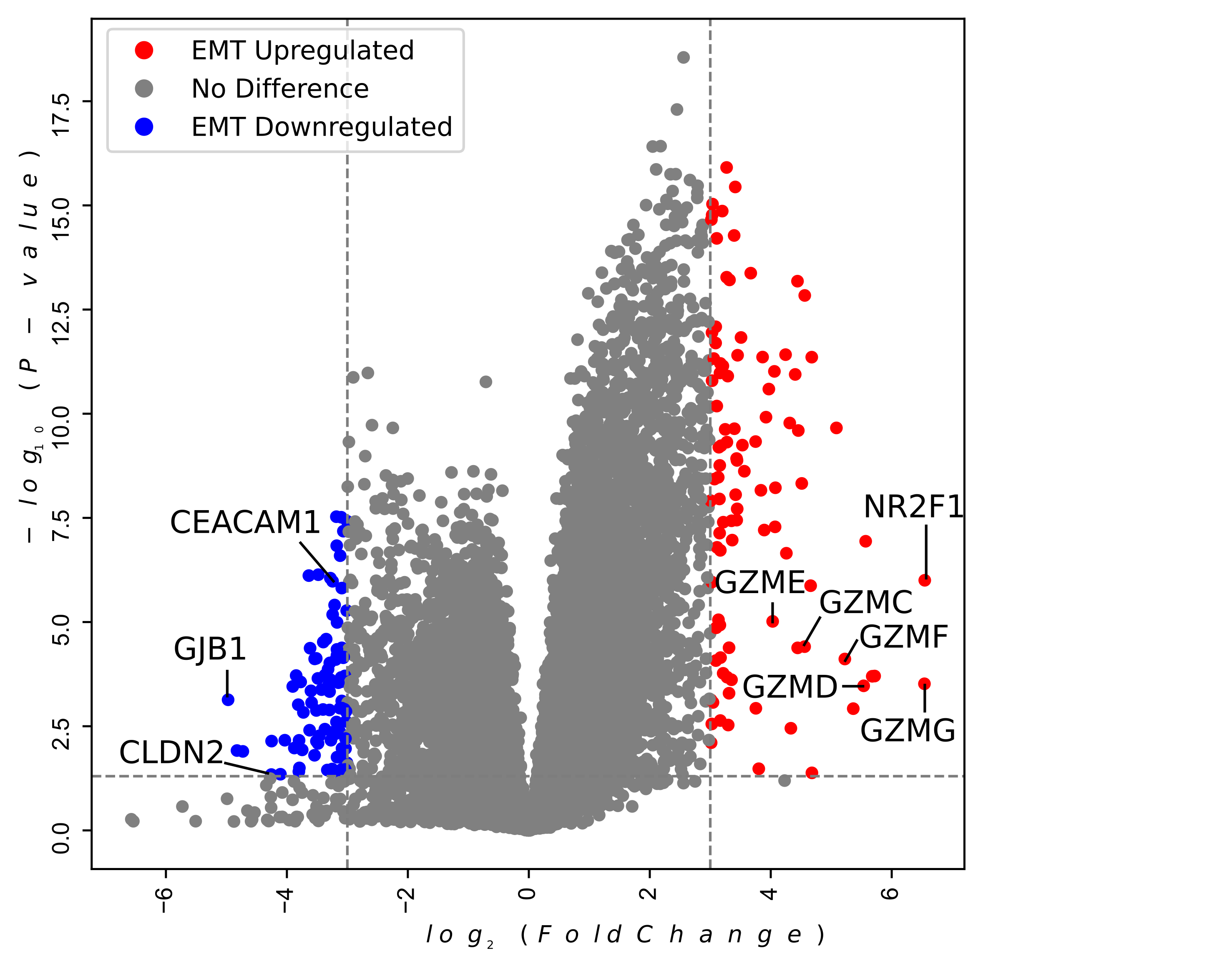

Supplement: Supplementary file 25 — Additional file 25: Fig. S21. Volcano plot of differentially expressed genes of MMTV-Myc EMT tumors compared to microacinar and squamous tumors. Log fold change is displayed along the x-axis and -log base 10 of the p-value determined by a students’ t-test is displayed along the y-axis. Log fold change cut off set at ≥ 3 and negative log base 10 cut off set at ≥ 1.3, which is equivalent to a p-value ≤ 0.05. Select genes are highlighted. The volcano plot is made using bioinfokit as a Python package. [file 13058_2023_1723_MOESM25_ESM.png]
